# Atrophic Kidney-Like Lesion – Case Report of A Provisional Entity with Brief Review of Literature

**DOI:** 10.5146/tjpath.2021.01541

**Published:** 2022-05-19

**Authors:** Balamurugan Thirunavukkarasu, Saket Singh, Aditya Prakash Sharma, Amanjit Bal

**Affiliations:** Departments of Histopathology, Post Graduate Institute of Medical Education & Research (PGIMER), Chandigarh , India; Departments of Urology, Post Graduate Institute of Medical Education & Research (PGIMER), Chandigarh , India

**Keywords:** Atrophic kidney-like lesion, Thyroid-like follicular carcinoma, Kidney

## Abstract

Atrophic kidney-like lesion is a recently recognized entity, post 2016 World Health Organization Classification of tumors of the urinary system. The behavior of this tumor is not fully known as only a handful of cases with limited follow-up are available. This entity closely mimics thyroid-like follicular carcinoma of the kidney, which has different prognosis.

We report a case of incidentally detected atrophic kidney-like lesion in an elderly gentleman who had urothelial carcinoma of the urinary bladder with a brief review of literature.

Atrophic kidney-like lesion and urothelial carcinoma of the urinary bladder association has not been reported in the literature.

## INTRODUCTION

Atrophic kidney-like lesion (AKLL)** **is a recent entity described post 2016 World Health Organization (WHO) classification of tumors of the urinary system. With the available limited follow-up data, this is considered as a benign renal neoplasm with indolent behavior. Owing to its follicular architecture, it closely resembles thyroid-like follicular carcinoma of the kidney (TFRCC) (1). The distinction between the two is essential as the latter has chromosomal alterations in the form of gains and losses and an aggressive behavior with metastatic potential (2,3).

## CASE REPORT

**Figure 1:**
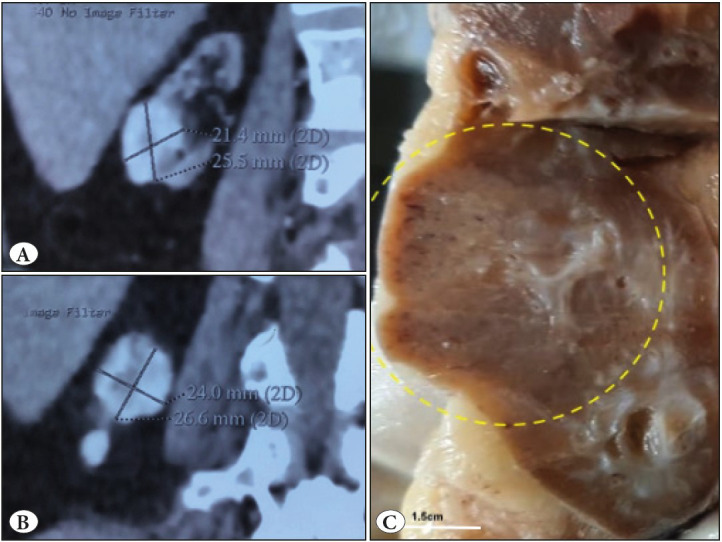
**A, B)** Contrast-enhanced CT abdomen showed a well-circumscribed tumour nodule in the upper pole in the background of a small kidney **C)** Cut surface shows a well-demarcated tan brown colored tumour measuring 3x2.8x2.5cm in the mid-third region.

**Figure 2:**
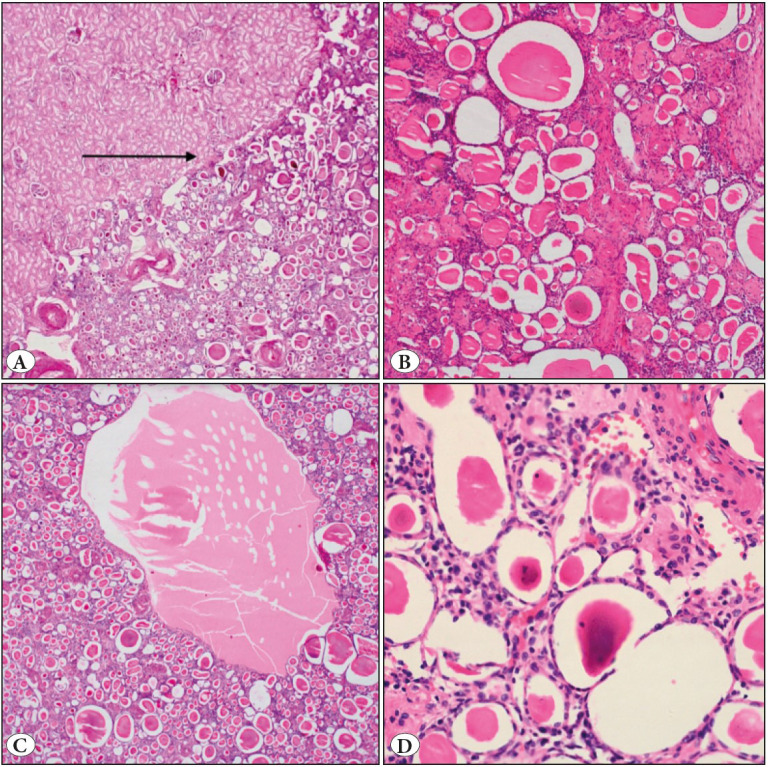
**A)** Tumour composed of multiple tightly packed varying sized follicles separated from native kidney by a thin capsule (arrow pointed) (H&E, 40x) **B)** The follicles are filled with dense eosinophilic colloid-like secretions (H&E, 100x) **C)** The lumen is filled with pink eosinophilic material and focal macrocystic degeneration (H&E, 40x) **D)** The follicles are lined by flattened to cuboidal epithelium with occasional amphophilic calcific deposits (H&E, 200x).

A 71-year-old gentleman was a known case of recurrent low-grade urothelial carcinoma for 9 months for which he had undergone transurethral bladder tumor resection thrice. On routine surveillance, contrast enhanced computed tomography (CECT) abdomen showed a well-circumscribed tumour measuring 2.6x2.4cm in the mid-third region of the right kidney with focal extension into the upper pole. The kidney measured 5.5cm in length with indistinct corticomedullary junction (Figure 1A,B). The patient underwent simple nephrectomy for the lesion. On gross examination, the renal capsule was intact, and the cut surface showed a well-demarcated tan brown lesion measuring 3x2.8x2.5cm in the mid-third region. (Figure 1C). The renal sinus and ureter were unremarkable. On microscopy, the lesion was well demarcated from the renal parenchyma by a thin fibrous capsule (Figure 2A). The lesion was composed of compact tubules lined by flattened and atrophic epithelium interspersed by cyst-like follicles. Many of the follicles were filled with pale to dense eosinophilic material detached from the epithelium (Figure 2B,C). The stroma between the follicles showed collagen deposition, few atrophic tubules, and capillaries. Focal amorphous calcific areas were also noted (Figure 2D). No atypical features like mitosis, necrosis or high nuclear grade areas were noted.

**Figure 3:**
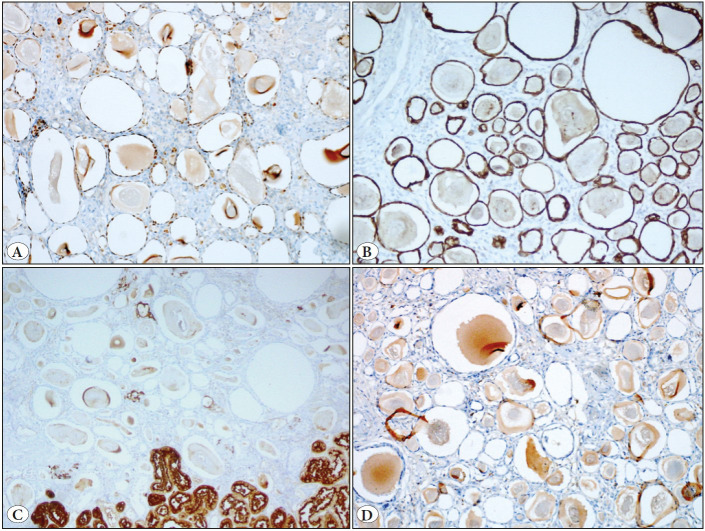
**A)** The tumour (follicular) cells are positive for PAX-8; **B)** CK7 Positive; **C)** CD10 Negative **D)** TTF-1 Negative (Immunoperoxidase, 200x).

The differential diagnoses considered were atrophic kidney-like lesion, thyroid follicle-like renal cell carcinoma, metastatic follicular carcinoma of the thyroid, and well-differentiated neuroendocrine tumour (carcinoid). A panel of immunohistochemistry was performed that included PAX8 (Cell Marque, RTU, clone MRQ-50), CK7 (Cell Marque, 1:300, clone OV-TL12/30), WT-1 (Dako, 1:50, clone 6F-H2), Synaptophysin (Cell Marque, 1: 200, Clone MRQ-40), CD117 (Dako, 1:500, clone A4502), and CD10 (Cell Marque, 1:30, clone 56C6). The cells were diffusely positive for PAX-8 (Figure 3A), and CK7 (Figure 3B), while negative for CD10 (Figure 3C), TTF-1 (Figure 3D), Synaptophysin, WT-1 and CD117. The renal sinus, pelvis and ureter were free.** **No extracapsular invasion was noted.** **Adjacent renal parenchyma showed preserved glomeruli and non-atrophic tubules. Based on morphology and immunophenotyping, an ‘Atrophic kidney-like lesion’ was diagnosed; Tumor stage – T1a; World Health Organization/International Society of Urological Pathology (WHO/ISUP) Nuclear grade 1. There were no post-surgical complications. The patient has been on regular follow-up till date without any recurrence or distant metastasis.

## DISCUSSION

The nomenclature “Atrophic kidney-like lesion (AKLL)” has been used interchangeably in the literature with “atrophic kidney-like tumour” and “atrophic kidney-like renal cell carcinoma” (4,5). This was originally described by Hes *et al* in 2014 as a separate entity and its clonal nature was proven in a series of 3 cases (1). Atrophic kidney-like lesion was initially considered under Thyroid-like follicular carcinoma of the kidney (TFRCC) (5). Following the original description, many cases were reviewed and described as reports and case series, of which few were initially diagnosed as TFRCC (6,7). The pathogenesis of AKLL is compared with that of glomerulocystic disease as the cells lining the cysts expressed WT-1, which is a marker of podocyte differentiation (8). At low power, both the tumors impart a follicular architecture. However, the distinguishing feature between AKLL and TFRCC can be appreciated on higher magnification. In TFRCC, the cells lining the follicles are cuboidal with abundant eosinophilic cytoplasm, high nuclear grade, and prominent nucleoli, resembling a proper follicular neoplasm of the thyroid whereas in AKLL the cells are flat/atrophic to low cuboidal (9,10).

Distinction of atrophic kidney-like lesion from the end stage renal disease induced by chronic pyelonephritis is important. Chronic pyelonephritis with thyroidization also shows atrophic tubules with hyaline casts mimicking the neoplasm. The important distinguishing point is the presence of a well-defined capsule, lack of inflammation, and absence of glomeruli in-between the lesion. In the present case, the background renal parenchyma did not show features of chronic pyelonephritis. The differential diagnosis of AKLL includes metastatic follicular carcinoma of the thyroid, metanephric adenoma, multilocular cystic renal neoplasm of low malignant potential, and tubulocystic renal cell carcinoma (2,11). Metastatic follicular carcinoma of the thyroid shows TTF-1 expression, whereas metanephric adenoma shows tightly packed tubules lined by uniform cuboidal cells with occasional presence of papillary structures and diffuse positivity for WT1. Multilocular cystic renal neoplasm of low malignant potential shows cystic areas lined by clear cells positive for CD10. Tubulocystic renal cell carcinoma shows uniformly dilated cystic spaces intervened by fibrotic to hyalinized stroma. The cystic spaces are lined by cells with significant nuclear abnormalities and variable hobnailing.

To the best of our knowledge, 13 cases of AKLL have been reported in the English literature (Table 1). Many of the cases were reviewed in the previously published cases of TFRCC. This lesion is usually reported in young patients with no major gender predilection. Age distribution of AKLL is from 9 to 58 years with a median age of 30.7 years. No specific genetic alterations have been reported yet (8). Classification of renal tumors is constantly evolving, and many new entities are being added and existing entities are being reclassified. Atrophic kidney-like lesion is a new entity with indolent behavior and described using different names. The Genitourinary Pathology Society (GUPS) has placed this lesion under “provisional entity” requiring more data for validation (4). The present case had thin well-defined capsule, occasional amorphous calcification with PAX8 and CK7 positivity. Features like high-grade nuclei, mitosis or necrosis were not seen. This index patient had completed treatment for recurrent low-grade papillary urothelial carcinoma of the urinary bladder and the renal lesion was incidentally detected on routine radiological surveillance. This association of AKLL with urothelial carcinoma has not been reported before.

**Table 1:** Published cases of atrophic kidney-like lesion in the literature.

**Author**	**Year**	**No. of cases**	**Age (yrs)**	**Comment**
Hes et al. (1)	2014	3	31.3 (mean)	Monoclonal nature proved in one case
Oshiro et al. (6)	2014	1	19	Bilateral tumors
Berens et al. (7)	2014	1	58	Incidentally detected at autopsy
Muscara et al. (10)	2017	1	27	Initially reported as TFRCC
Herlitz et al. (8)	2018	6	29 (mean)	Elucidation of pathogenesis akin to glomerulocystic kidney disease
Amin et al. (9)	2009	1	29	One case initially reported as TFRCC
Present case	2021	1	71	Incidentally detected post urothelial carcinoma of the bladder

**TFRCC: **Thyroid-like follicular carcinoma of the kidney

## Conflict of Interest

The authors declare no conflict of interest.
